# Risk factors associated with asthma exacerbations in school-age children

**DOI:** 10.1186/2045-7022-5-S2-P6

**Published:** 2015-03-23

**Authors:** Tolga S Yavuz, Ozgur Kartal, Guven Kaya, Mustafa Gulec, Mehmet Saldir, Osman Sener

**Affiliations:** 1 GATA School of Medicine, Department of Pediatric Allergy, Ankara, Turkey; 2 GATA School of Medicine, Department of Adult Allergy, Ankara, Turkey; 3 GATA School of Medicine, Department of Pediatrics, Ankara, Turkey

## Background

Acute asthma exacerbation is one of the most frequent emergencies in childhood. Awareness of risk factors related with asthma exacerbation will reduce the morbidity and the mortality of the disease. The aim of this study is to investigate the factors associated with asthma exacerbations in school-age children.

## Method

Children who attended to a tertiary outpatient pediatric allergy and asthma department and diagnosed with asthma were enrolled in the study. A questionnaire including demographic features and parameters to determine socioeconomic status were applied. Asthma control status of patients was evaluated according to GINA criteria. Laboratory investigations including complete blood counts with differential, total IgE levels, skin prick tests and pulmonary function tests were also performed.

## Results

A total of 348 children (232 male (66.7%); with a median age [interquartile range] of 8.0 [6.2-11.1] years were included. 45.9% of children had aeroallergen sensitization. Asthma was controlled in 138 children (39.7%), whereas partially controlled and uncontrolled in 45 (12.9%) and 98 (28.2%) patients, respectively. 67 patients (19.3%) were having an asthma exacerbation. The history of ER admittance and asthma exacerbation in the last year was more frequent in children with asthma exacerbation (p <0.001 and p<0.001, respectively). Moreover, children with asthma exacerbation were less frequently under regular asthma controller therapy (p<0.001). Multivariate logistic regression analysis revealed that history of asthma exacerbation in the last year (Odds Ratio [Confidence Interval]) (22.47 [9.28-54.44]; p<0.001), lack of regular asthma controller therapy (3.51 [1.22-10.10]; p=0.02), lack of previous asthma diagnosis (3.34 [1.70-6.57]; p< 0.001) and being overweight (2.25 [1.15-4.40]; p=0.018) were related with asthma exacerbation in school-age children with asthma.

## Conclusion

Awareness of risk factors related with asthma exacerbation may alert physicians who deal with school-age children with asthma and may help prompt and rational interventions in order to prevent asthma exacerbations.

